# Slow myosin heavy chain 1 is required for slow myofibril and muscle fibre growth but not for myofibril initiation

**DOI:** 10.1016/j.ydbio.2023.04.002

**Published:** 2023-07

**Authors:** Hoi-Ting A. Hau, Jeffrey J. Kelu, Julien Ochala, Simon M. Hughes

**Affiliations:** a Randall Centre for Cell and Molecular Biophysics, School of Basic and Medical Biosciences, King's College London, SE1 1UL, UK; b Centre for Human & Applied Physiological Sciences, School of Basic and Medical Biosciences, King's College London, SE1 1UL, UK

## Abstract

Slow myosin heavy chain 1 (Smyhc1) is the major sarcomeric myosin driving early contraction by slow skeletal muscle fibres in zebrafish. New mutant alleles lacking a functional *smyhc1* gene move poorly, but recover motility as the later-formed fast muscle fibres of the segmental myotomes mature, and are adult viable. By motility analysis and inhibiting fast muscle contraction pharmacologically, we show that a slow muscle motility defect persists in mutants until about 1 month of age. Breeding onto a genetic background marking slow muscle fibres with EGFP revealed that mutant slow fibres undergo terminal differentiation, migration and fibre formation indistinguishable from wild type but fail to generate large myofibrils and maintain cellular orientation and attachments. In mutants, initial myofibrillar structures with 1.67 ​μm periodic actin bands fail to mature into the 1.96 ​μm sarcomeres observed in wild type, despite the presence of alternative myosin heavy chain molecules. The poorly-contractile mutant slow muscle cells generate numerous cytoplasmic organelles, but fail to grow and bundle myofibrils or to increase in cytoplasmic volume despite passive movements imposed by fast muscle. The data show that both slow myofibril maturation and cellular volume increase depend on the function of a specific myosin isoform and suggest that appropriate force production regulates muscle fibre growth.

## Highlights

• Knockout of *smyhc1* yields viable zebrafish that recover from a mild early motility defect.
• Residual myofibrils with sarcomeric myosin immunoreactivity are present in slow muscle fibers.
• The absence of Smyhc1 greatly reduces the extent of myofibril formation and reduces larval slow fiber volumetric growth.

## 1 Introduction

Myosin are motor proteins that drive actin-based motility (Szent-Gyorgyi, 2004). There are more than 35 classes of myosin, each consisting of a myosin heavy chain (MyHC) and a number of smaller proteins, each class being defined by its distinct structure and function (Thompson and Langford, 2002; Sweeney and Houdusse, 2010). Class II myosins are found across metazoa, arose early in eukaryotic evolution (Richards and Cavalier-Smith, 2005, Thompson and Langford, 2002) and are composed of a heterohexamer containing two motor domains each made from a MyHC subunit, bound to a pair of non-identical myosin light chains (MyLCs) and linked by a coiled-coil MyHC tail that attaches to cargo. The sarcomeric myosins of striated muscle are a subset of class II MyHCs that form the regular filamentous array of thick myosin filaments interdigitating with parallel arrays of thin actin filaments (Hall et al., 1946; Hanson and Huxley, 1953). These structures are stabilised by M-lines and Z-lines that crosslink myosin thick filaments and actin thin filaments respectively, to form a sarcomere, many of which are linked in series to form a myofibril (Alberts et al., 2014, Howard, 1997). Myosin motor function within the bipolar arrays exerts force on actin thin filaments, shortening the sarcomeres, and thereby the myofibril, bringing about muscle contraction.

Structurally, a sarcomeric class II MyHC molecule has a conserved N-terminal motor head domain, which has actin-activated ATPase activity, followed by a rod region. The rod has a lever arm (containing the two MyLC binding sites), followed by a helical light meromyosin (LMM) region that dimerizes to form a coiled-coil, which then aligns with other heterohexamers to form a thick filament. The cooperative properties of these molecules within the myofibril determine the contractile characteristics of the muscle fibre, in particular its force/velocity relationship and energy efficiency (Irving, 2017).

Mammalian skeletal muscle fibres can be divided into slow-twitch and fast-twitch fibre types. Slow-twitch type I fibres contain slow MyHC (encoded by the *MYH7* gene) which gives slow fibres a low maximal shortening velocity. Fast-twitch type II fibres generally contain MyHCs with one of three progressively faster maximal shortening velocities (IIa, IIx and IIb, encoded by *MYH2*, *MYH1* and *MYH4*, respectively). Coordinated metabolic properties give slow-twitch fibres oxidative metabolism and slow activation and relaxation rates, whereas the faster fibres have glycolytic metabolism and are rapidly activated/inactivated (Bottinelli, 2001, Schiaffino and Reggiani, 2011, Weiss et al., 2001). A number of murine adult fast MyHC genes have been genetically ablated, but lead to rather mild physiological phenotypes arising after 6 weeks of age that appear to be compensated by upregulation of other fast MyHC genes (Acakpo-Satchivi et al., 1997; Allen and Leinwand, 2001). Deletion of earlier developmental fast MyHC isoforms, on the other hand, can have stronger effects (Agarwal et al., 2020), https://www.mousephenotype.org/data/genes/MGI:1339712. Thus, the importance of individual fast MyHC genes depends on details of their function/s that are not immediately apparent from their known biochemistry.

Mammals also employ slow MyHC in cardiac muscle. *MYH7* encodes the so-called β-cardiac slow MyHC that is expressed by ventricular cardiomyocytes. A genetically-linked duplicate gene, *MYH6*, expresses α-cardiac MyHC in adult atrial cardiomyocytes, conferring upon them distinct contractile properties (Alpert et al., 2002). Homozygous mutation of *MYH6* or *MYH7* slow MyHCs is lethal in mice due to early heart defects (Gifford et al., 2019; Jones et al., 1996). In contrast, heterozygous missense mutations in either gene are known to cause cardiac disease in humans that can be modelled in mice (Blankenburg et al., 2014; Gifford et al., 2019). To date, analysis of the effect of loss of slow MYHC function specifically in mammalian skeletal muscle has not been performed.

Slow myosins in non-mammalian vertebrates have undergone distinct gene duplication events, but are, nevertheless, found to be expressed primarily in cardiac and slow skeletal muscle (www.zfin.org). In zebrafish, one genomic locus contains at least four tandemly-arrayed *smyhc* genes that are not expressed in the heart (Elworthy et al., 2008; McGuigan et al., 2004). Elsewhere in the zebrafish genome, various other genes with greatest homology to mammalian *MYH6/7* exist (named *myh6*, *myh7*, *myh7l*, *myh7ba*, and *myh7bb*) and are expressed in heart and/or skeletal muscle (Berdougo et al., 2003; Shih et al., 2015; Thisse and Thisse, 2004; Wang et al., 2011). *Smyhc1* is the primary gene expressed in the earliest slow skeletal muscle of the zebrafish (Bryson-Richardson et al., 2005; Elworthy et al., 2008; Thisse and Thisse, 2004), which is the first functional muscle. Mammals also express slow myosin in their earliest muscle fibres (Kelly and Rubinstein, 1980).

Zebrafish slow muscle fibres derive from adaxial cells that undergo terminal differentiation in the medial somite, prior to migrating to the lateral myotome surface as fast muscle fibres form behind them (Blagden et al., 1997; Devoto et al., 1996; Hinits and Hughes, 2007). In each myotome, approximately 20 mononucleate slow fibres align anteroposteriorly and gradually increase in number as the myotome grows by fibre addition at the dorsal and ventral myotomal extremes (Barresi et al., 2001). Subsequently, slow fibres increase in size, maturing to form a specialised slow muscle with a uniform fibre type at the lateral midline (Rowlerson and Veggetti, 2001). Biomechanical arguments suggest that the positioning of oxidative slow fibres in this location enables continuous low-speed swimming at minimal energetic cost (Videler, 1993). The importance of the Smyhc1 protein for each of these developmental, cell biological and physiological processes is poorly understood.

Here we describe generation and characterisation of new loss of function *smyhc1* mutants, which show significant differences in phenotype from some *smyhc1* mutant alleles previously published. Mutant fish are viable and fertile but show a slow muscle specific motility defect during early life that recovers in the late larva at the time the *smyhc2* gene is normally induced. The embryonic slow muscle fibres in mutants are formed, migrate, align and assemble immature myofibril-like structures containing other MyHC molecules, but fail to build an abundant array of large myofibrils or correctly increase cytoplasmic volume to form a fibre of the normal size and shape. We conclude that specific MyHC genes are required not only for appropriate motility, but also for the correct assembly of muscle fibre structure.

## 2 Results

### 2.1 Generation of *smyhc1* mutant alleles

**Fig. 1:**
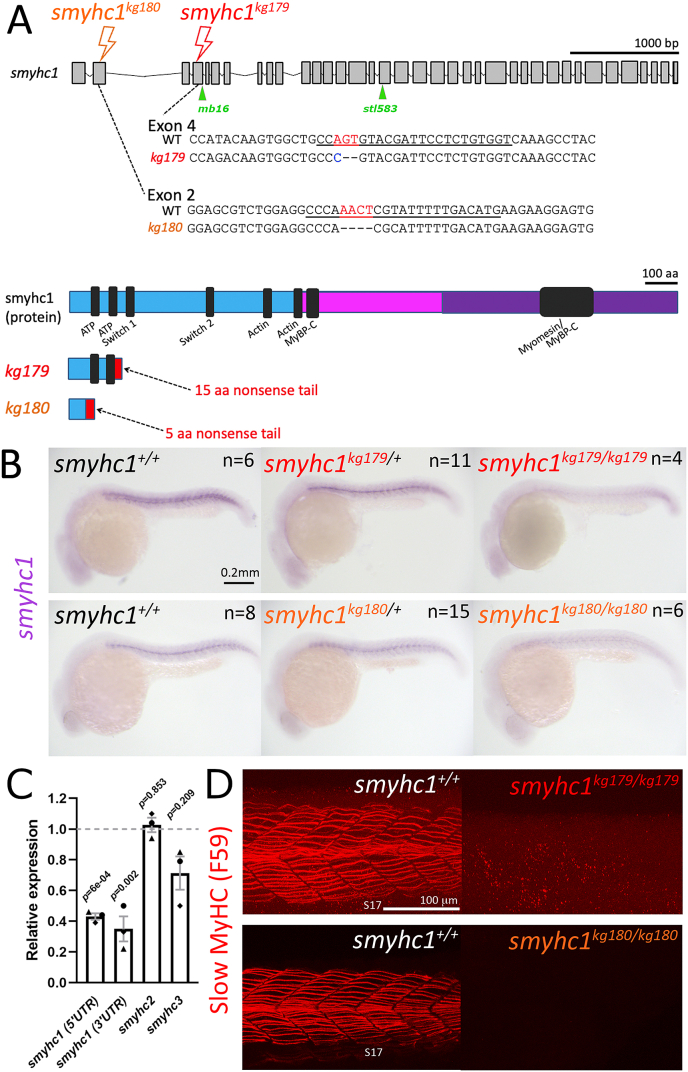
**Genome editing generates likely null alleles of zebrafish *smyhc1.*** (A) Schematic of *smyhc1* gene, mRNA sequences and proteins showing nature of *smyhc1*^*kg179*^ and *smyhc1*^*kg180*^ mutant alleles. Underline indicates the guide RNA sequence, red and blue bases were deleted and inserted, respectively. Most of the S1 (cyan) and all the S2 (magenta) and LMM domains (purple) of the protein are lost in the mutants, ablating most highly-conserved regions (black). Location of other *smyhc1*^*mb16-18*^ and *smyhc1*^*stl583*^ truncation alleles are marked (green). (B) *In situ* RNA hybridisation for *smyhc1* mRNA in *smyhc1* mutant and their siblings from *smyhc1*^*kg179/+*^ and *smyhc1*^*kg180/+*^ in-crosses reveal nonsense-mediated decay of mutant *smyhc1*^*kg179*^ and *smyhc1*^*kg180*^ mRNA at 24 hpf. In a randomly-selected sample from a *smyhc1*^*kg179/+*^ incross, 4/21 had reduced expression and were shown to be mutant; among 17 ‘normal’ expressors 11/20 were heterozygotes and 6/20 were wild type upon sequence genotyping. From the *smyhc1*^*kg180/+*^ incross, 6/29 embryos had low expression and were shown to be mutant; among 23 normal expressors 15/23 were heterozygous and 8/23 were wild type upon sequence genotyping. (C) Quantitative RT-PCR for *smyhc1*, *smyhc2* and *smyhc3* on pools of ten 2 dpf embryos from a *smyhc1*^*kg179/+*^ incross that had been sorted into immotile mutant and motile siblings at 1 dpf. Each symbol represents a separate reverse transcription with symbol shapes distinguishing biological replicate lays from separate parents. The dashed line represents the motile sibling level (D). Maximum intensity projections of Slow MyHC-stained 24 hpf embryos showing region centred on somite 17 (S17). Wild-type siblings (left) show slow fibre staining and *smyhc1*^*kg179/kg179*^ and *smyhc1*^*kg180/kg180*.^mutant siblings show no slow fibre stain (right).

To generate *smyhc1* mutants, CRISPR/Cas9 genome editing with distinct gRNAs was used to target *smyhc1* in the first and third coding exons. Several mutant alleles were generated, among these mutations *smyhc1*^*kg179*^ and *smyhc1*^*kg180*^ were chosen for analysis. *Smyhc1*^*kg179*^ contains a 3 bp deletion and 1 bp insertion in exon 4 leading to a frameshift at amino acid 134 and an early stop codon at amino acid 148 after a 15 amino acid nonsense tail (Fig. 1A). *Smyhc1*^*kg180*^ contains a 4 bp deletion in exon 2 leading to a frameshift at amino acid 28 and an early stop codon at amino acid 32 after a 5 amino acid nonsense tail (Fig. 1A). *Smyhc1*^*kg179*^ lacks most highly-conserved motifs including most of the amino acids of the ATP binding site. *Smyhc1*^*kg180*^ lacks all highly-conserved motifs. Each *smyhc1* mutant line was outcrossed to wild-type AB fish at F1 and F2 and first bred to homozygosity at F3. Lays (spawnings) from *smyhc1*^*kg179/+*^ and *smyhc1*^*kg180/+*^ incrosses were examined for morphological and skeletal muscle defects. Consistent with previous studies using antisense morpholino to *smyhc1* (Codina et al., 2010; Xu et al., 2012) at 1 dpf homozygous mutants for both *kg179* and *kg180* appeared immotile, but morphologically normal (Fig. 1B; 86/344 (25%) and 12/52 (23%) immotile embryos, respectively)*.* Incross between heterozygotes of each line also yielded immotile embryos at the expected transheterozygote frequency (5/19 molecularly-genotyped larvae (26%)), showing that off-target effects did not cause immotility.

**Supplementary Fig. 1:**
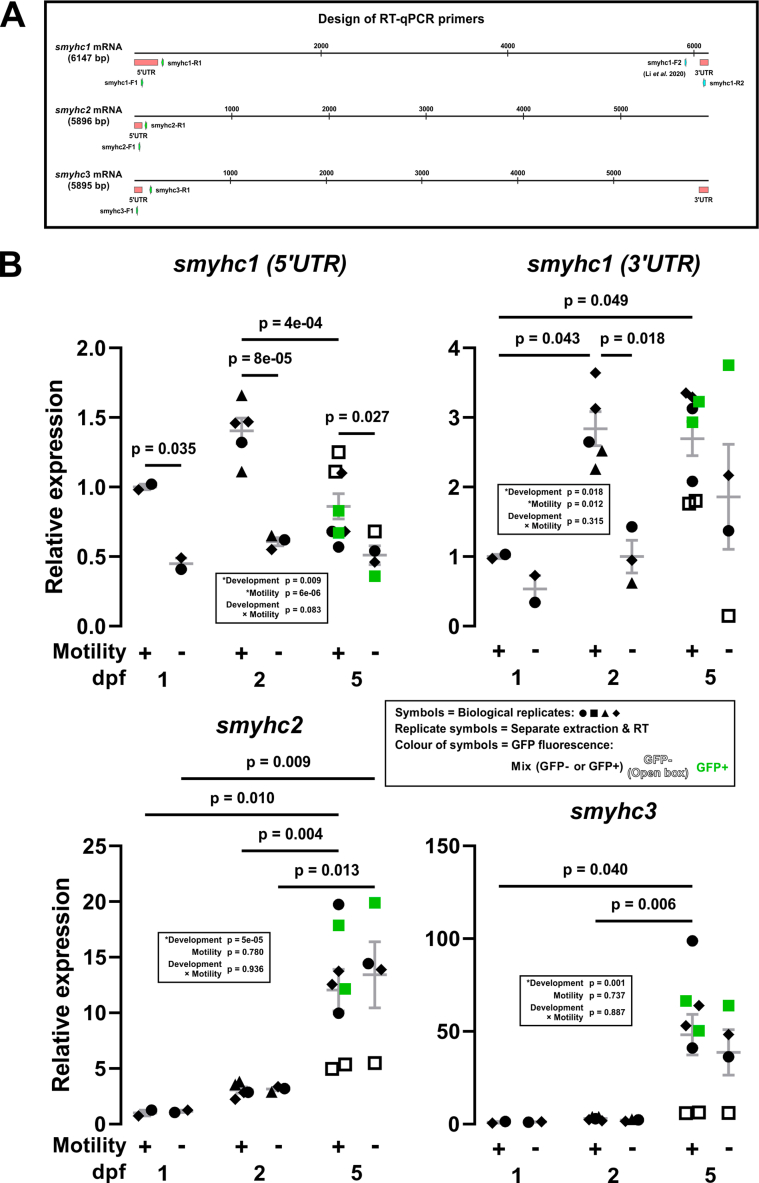
Nonsense-mediated decay of *smyhc1* mRNA and lack of compensatory upregulation by *smyhc2* or *smyhc3* mRNAs. (A) Schematic showing the design of RT-qPCR primers for *smyhc1-3* transcripts. To ensure specificity, primer sets were designed to target the 5′ or 3′ untranslated region (UTR) of the transcripts. **(B)** Expression of *smyhc1-3* at 1, 2 and 5 dpf in siblings from *smyhc1*^*kg179/+*^*;Tg(smyhc1:EGFP)*^*i104/+*^ in-crosses that had been sorted at 1 dpf into motile and immotile groups. Total RNA prepared from 5 or 10 embryos/larvae per sample from two to four independent lays (represented by symbol shape). Some embryos/larvae were further sorted based on EGFP fluorescence (represented by symbol colour). Bars show mean ​± ​SEM of all samples. Statistics are two-way ANOVA (Stage/Motility) with Bonferroni's post hoc test.

Whole mount in situ mRNA hybridisation and quantitative RT-PCR showed that nonsense-mediated mRNA decay of *smyhc1* mRNA occurs in mutants (Fig. 1B and C). Whereas *smyhc1* mRNA was readily detected in slow muscle of sibling embryos at 24 hpf, much less signal was observed in about a quarter of embryos, the genotype of which were subsequently confirmed as *smyhc1*^*kg179*^ and *smyhc1*^*kg180*^ homozygous mutants (Fig. 1B). Similarly, immotile embryos contained less *smyhc1* mRNA than motile siblings (Fig. 1C, Supplementary Fig. 1). Thus, the mutations cause premature ribosomal termination leading to mRNA decay and therefore little mutant protein will be produced. Indeed, mutants lacked detectable Smyhc1 protein, whereas their siblings expressed in approximately 20 superficial slow fibres (SSFs) and several fibres deeper in the horizontal myoseptum, the slow muscle pioneers (Fig. 1D). We conclude that *smyhc1*^*kg179*^ and *smyhc1*^*kg180*^ are likely strong loss of function, and possible null, alleles.

### 2.2 *smyhc1* mutants are viable and fertile

**Fig. 2:**
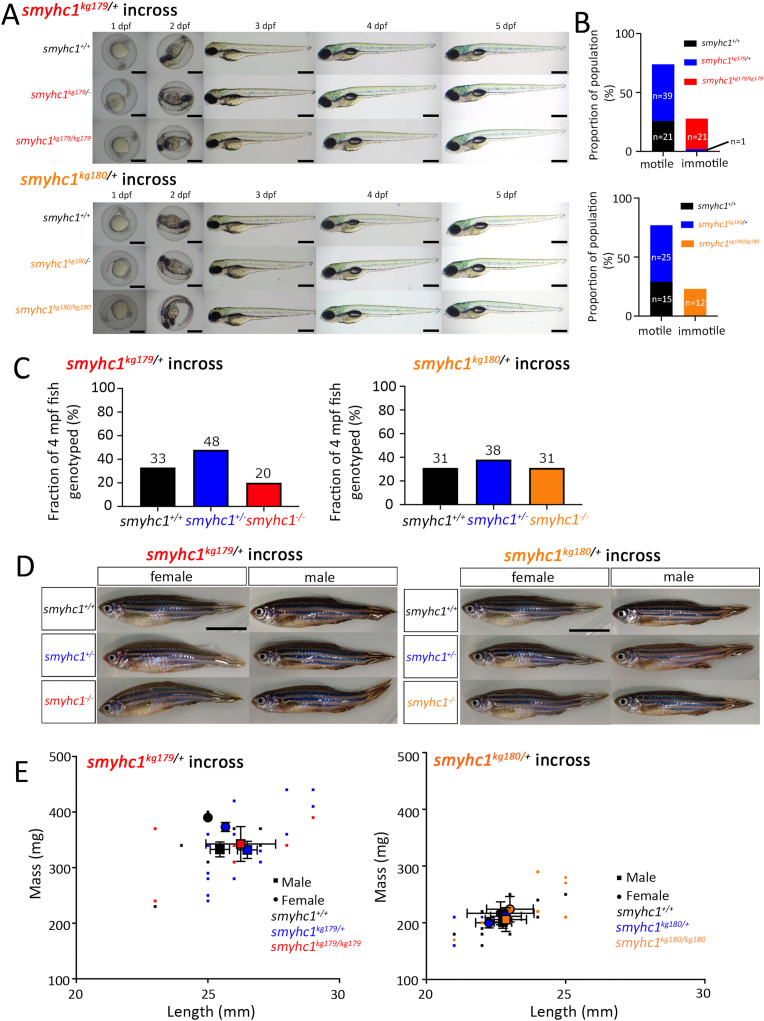
**Zygotic *smyhc1* mutation reduces embryo motility but survive to adulthood. A)** Representative bright-field images of 1,2,3,4 and 5 dpf larvae from *smyhc1*^*kg179/+*^ and *smyhc1*^*kg180/+*^ heterozygote in-crosses. Fish are shown anterior towards the left and dorsal upwards with genotyped heterozygotes and mutants below their respective wild type siblings. Scale bars ​= ​0.5 ​mm. **B)** Randomly selected larvae were dechorionated at 24 hpf and examined for presence or absence of tail coiling movement in *smyhc1*^*kg179/+*^ in-cross (n ​= ​82) and in *smyhc1*^*kg180/+*^ in-cross (n ​= ​52). The genotype of the fish was determined after the examination by DNA sequencing. Fish derived from several in-crosses of *smyhc1*^*kg179/+*^ (n ​= ​101) and *smyhc1*^*kg180/+*^ (n ​= ​100) fish were reared with their siblings and genotyped at 4 months post-fertilization (mpf). **C)** Adults showed the expected Mendelian ratios at 4 mpf. Fish numbers above each bar. **D)** Adults at 12 mpf. Scale bars ​= ​1 ​cm. **E)** Length and mass at 4 mpf of genotyped siblings showed no significant difference between genotypes. Small symbols indicate individuals, large symbols the mean for each sex and genotype ​± ​S.E.M. Overall length and weight were less in adult fish from the *smyhc1*^*kg180/+*^ lay compared to adult fish from the *smyhc1*^*kg179/+*^ lay, a difference that may reflect an uncontrolled environmental or genetic background effect.

**Fig. 3:**
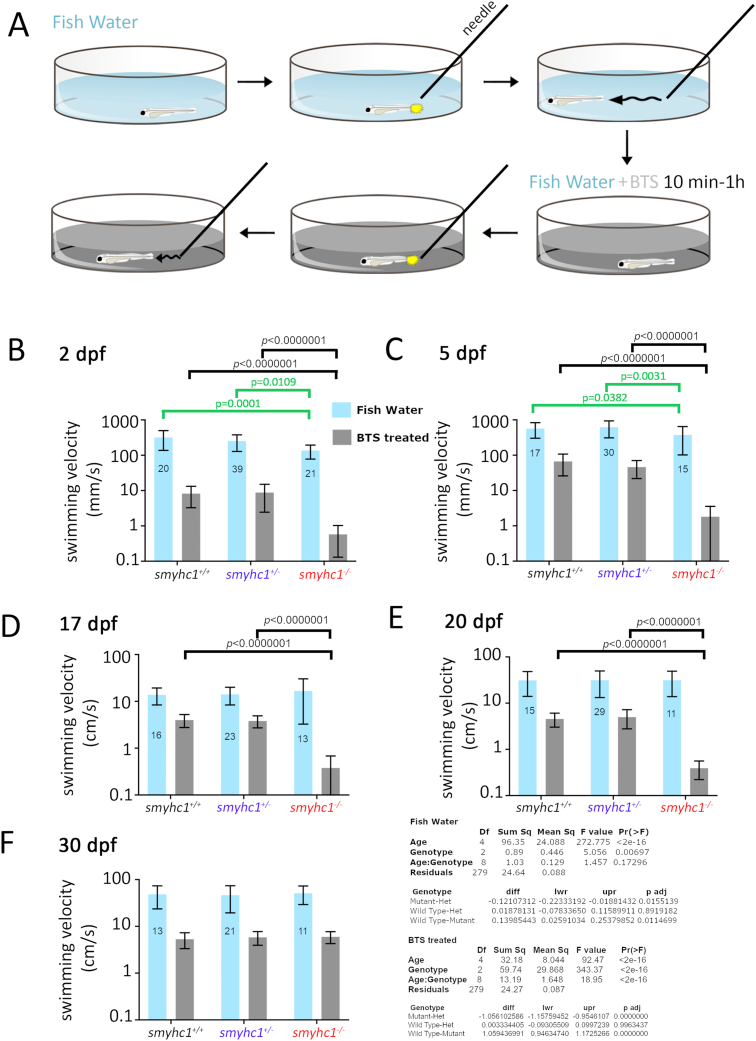
**Loss of Smyhc1 reduces swimming velocity.** Zebrafish larvae from *smyhc1*^*kg179/+*^ in-crosses in fish water were assayed for swimming activity upon touch stimulation with a pipette before and then after transient treatment with 50 ​μM fast myosin inhibitor BTS. **A)** Schematic describing workflow. **B–F)** Quantification of velocity (mean ​± ​SD, N ​= ​indicated on bars) on fish of indicated ages were obtained using at least three separate lays from heterozygous *smyhc1*^*kg179/+*^ in-crosses. Full dataset is in Table S1. Note the log_10_ scale on the *Y*-axes to show both control Fish Water and BTS-treated data accurately. Summary Tukey *post hoc* statistics for separate two way ANOVAs on Fish Water and BTS-treated are shown below. In each case, the upper table shows the overall significance, whereas the lower table shows the Tukey *post hoc* significance levels for the overall effects of genotype independent of age. Values of within-age Tukey *post hoc* test comparisons are indicated above graphs (black lines and *p*-values), none of which had *p* ​< ​0.05 in Fish Water. To mitigate the effect of changing variance due to the increase in velocity with age and large effect of BTS, individual one way ANOVAs on genotype were performed at each age and Tukey *post hoc* tests supported the reduced velocity of mutants in Fish Water at 2 and 5 dpf only (green lines and *p*-values).

Lays from *smyhc1*^*kg179/+*^and *smyhc1*^*kg180/+*^ in-crosses were sorted based on immotility at the 23 somite stage (23ss) and examined under a bright-field microscope at 1 to 5 dpf to identify any morphological and skeletal muscle defects. Mutant larvae were detected at the expected frequency, indicating that null mutations in *smyhc1* are not embryonically lethal. In many separate in-cross lays of *smyhc1*^*kg179/+*^, one lay from *smyhc1*^*kg180/+*^, and one transheterozygote lay, no change in head, somite, tail, yolk sac, fin, pigmentation, or body length was observed (Fig. 2A). However, as previously reported in studies of antisense morphants and distinct *smyhc1* mutants (Codina et al., 2010; Li et al., 2020; Whittle et al., 2020; Xu et al., 2012), for both *kg179* and *kg180* immotile embryos were genotyped as mutant at 24 hpf (Fig. 2B; 21/22 and 12/12 immotile embryos, respectively)*.* By 34 hpf, however, all embryos were motile irrespective of genotype, explaining the ability of mutants to hatch by 3 dpf and swallow air and inflate the swim bladder by 4 dpf (Fig. 2, Fig. 3). Thus, lack of Smyhc1 in *kg179* and *kg180* mutants leads to fish that appear immotile at early stages but morphologically normal.

To determine whether *smyhc1* mutation affects survival beyond 5 dpf and into adulthood, F3 embryos generated from *smyhc1*^*kg179/+*^ and *smyhc1*^*kg180/+*^ in-crosses were reared. One hundred randomly-selected embryos from each cross were monitored for 4 months. Growth of all siblings from crossed fish were divided in tanks of 50 larvae of mixed sex and genotype to ensure competition. At 4 months post-fertilisation (mpf), 82% and 94% survival was observed from *smyhc1*^*kg179/+*^ and *smyhc1*^*kg180/+*^ crossed fish, respectively. Genotyping of a randomly-selected subset of 4 mpf adult fish revealed that both lays did not differ from expected Mendelian ratios (Fig. 2C, *p* ​> ​0.05 Χ^2^-tests), although *kg1*80 had fewer heterozygotes than expected (*p* ​= ​0.016). Mutants remained indistinguishable from siblings during later life (Fig. 2D). Length and weight measurements were taken on mutant fish reared with their siblings at 4 mpf. Fish from the *smyhc1*^*kg180/+*^ incross were overall smaller in length and weighed less than fish from the *smyhc1*^*kg179/+*^ incross, possibly reflecting uncontrolled genetic background or, perhaps more likely, environmental rearing conditions. Nevertheless, when comparing between siblings, no significant difference in length or weight was observed between sex-matched wild-type, heterozygote or mutant fish (Fig. 2E). Homozygous *smyhc1*^*kg179/179*^ and *smyhc1*^*kg180/kg180*^ mutant males and females were fertile. We conclude that *smyhc1* is a non-essential gene for life in an aquarium.

### 2.3 Movement defects persist in *smyhc1* mutant

We next determined whether the defective movement in *smyhc1* mutants persists beyond 24 hpf. Previous studies have shown immotility in zebrafish at 24 hpf in *smyhc1* morphants or mutants (Codina et al., 2010; Li et al., 2020; Whittle et al., 2020; Xu et al., 2012), when movements are primarily driven by early-formed slow fibres (Drapeau et al., 2002). *Smyhc1*^*kg179/+*^ were in-crossed to generate wild type, heterozygous and homozygous mutant embryos. Microscopical observation of anterior somites at or shortly after 24 hpf revealed minor twitching movements in mutants, likely driven by nascent fast fibres, which do not express *smyhc1*, but begin to assemble striated myofibrils at this stage (Hinits and Hughes, 2007). Chorions were removed at 24 hpf and tail-coiling movement was analysed to separate motile from immotile fish, and their movement assayed over subsequent days (Fig. 3A). At 48 hpf, immotile mutants had regained tail muscle motility and superficially appeared to move similarly to wild-type and heterozygous siblings. Nevertheless, when swimming velocity upon touch stimulation was assayed, homozygous *smyhc1*^*kg179/kg179*^ mutants showed reduced swimming velocity (136 ​mm ​s^−1^) compared to their wild type (*p* ​= ​0.011) and heterozygous siblings (285 ​mm ​s^−1^; *p* ​= ​0.016) (Fig. 3B). At 5 dpf, *smyhc1*^*kg179/kg179*^ mutants had reduced swimming velocity (375 ​mm ​s^−1^) compared to their wild-type and heterozygous siblings (543 ​mm ​s^−1^) (Fig. 3C). From 17 to 30 dpf, no difference between *smyhc1*^*kg179/kg179*^ and their siblings was apparent (Fig. 3D–F). Thus, loss of Smyhc1 results in reduced swimming capacity in young fish.

To examine motility driven by slow fibres, 2 dpf embryos were treated with 50 ​μM N-benzyl-p- toluene sulphonamide (BTS), an inhibitor for fast muscle myosin II (Cheung et al., 2002; Li and Arner, 2015) and their swimming velocity was recorded (Fig. 3B). All fish showed strongly reduced swimming velocity after treatment with BTS (Fig. 3B–F). However, at 2, 5, 17 and 20 dpf homozygous *smyhc1*^*kg179/kg179*^ mutants were more affected than their wild-type and heterozygous siblings, showing either little twitching or no movement (p ​< ​0.000001; Fig. 3B–E). At 30 dpf, BTS reduced swimming velocity to a similar extent as at 20 dpf, but there was no significant difference between homozygous *smyhc1*^*kg179/*kg179^ mutants and their siblings (Fig. 3F). Thus, slow fibre motility remains compromised in young mutant larvae, before recovering during the fourth week of life.

### 2.4 Defective sarcomere organisation in mutant slow fibres

**Fig. 4:**
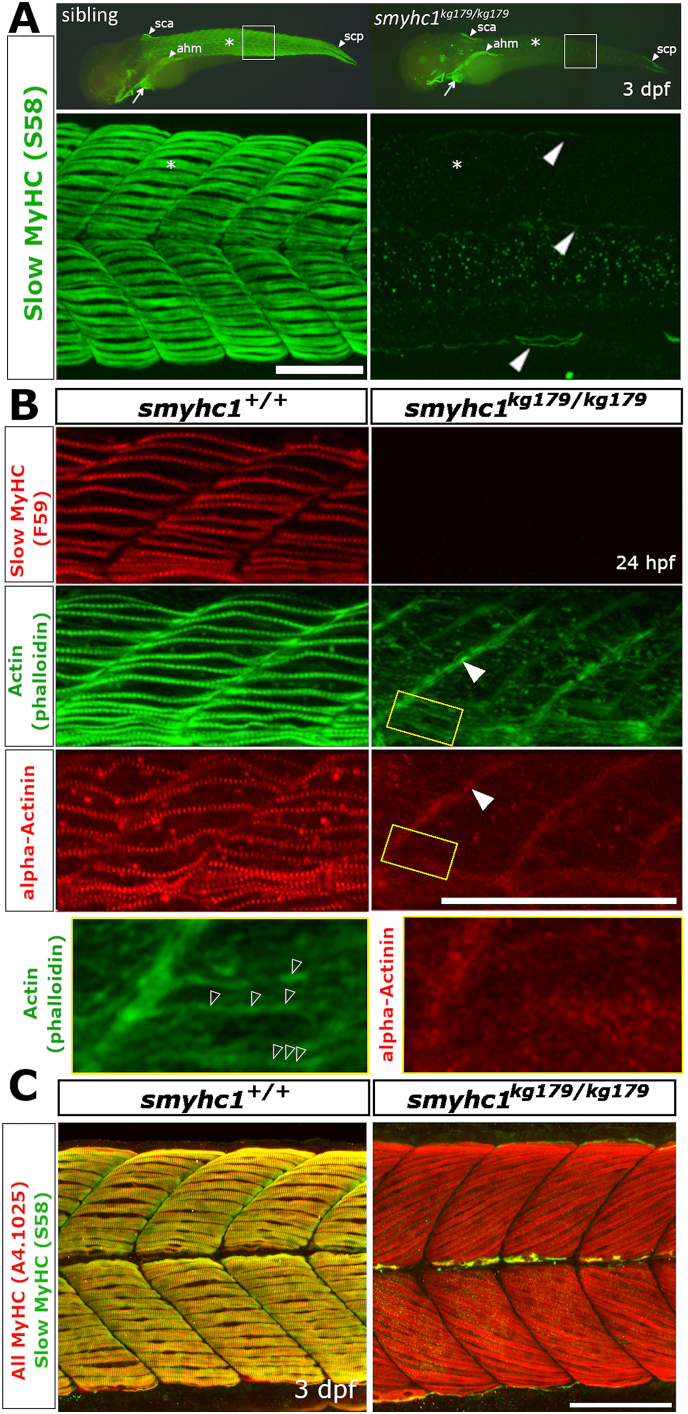
**Defective sarcomere organisation in *smyhc1***^***kg179/kg179***^**mutants.** Sibling zebrafish from *smyhc1*^*kg179/+*^ in-crosses mounted dorsal up, anterior to left, shown in wholemount (A, upper panel) or maximal intentisity projection confocal stacks. **A)** Slow MyHC immunofluorescent detection with antibody S58 at 3 dpf shows signal in superficial slow fibres (SSFs) of heterozygote sibling (n ​= ​8/8 sibs) but its absence in mutant (asterisks), despite the presence in mutant of signal in cranial, cardiac (arrows), and specific somite-derived muscle (arrowheads; n ​= ​6/6). White boxes are shown enlarged beneath to highlight S58 signal in thin nascent SSFs at dorsal and ventral somitic extremes and muscle pioneers at the horizontal myoseptum in mutant (arrowheads). **B)** Immunodetection of slow MyHC with antibody F59 and α-actinin to mark Z-disks and phalloidin staining to reveal filamentous actin in somite 17/18 region at 24 hpf. Note the absence of organised Z-lines and thin filament arrays in the mutant but the persistence of signal at the verticle myoseptum (filled arrowheads). Boxed areas magnified beneath show repeated F-actin structures in mutant (open arrowheads) that appear to lack α-actinin. **C)** Immunodetection of slow MyHC with antibody S58 and all sarcomeric MyHC with A4.1025 reveals lack MyHC in mutant slow fibres but presence in underlying fast fibres in somite 16–19 region at 3 dpf. Note the poor binding of A4.1025 to fast fibres in the wild type, presumably due to adsorption to slow fibres. Abbreviations: ahm – anterior hypaxial muscle, sca - supracranialis anterior, scp - supracranialis posterior. Bars ​= ​100 ​μm.

To understand the basis for motility defects and recovery in *smyhc1* mutants, we next investigated sarcomere assembly in specific muscle cell populations (Fig. 4). Using antibody S58, which is known to detect specifically slow MyHC isoforms in a variety of species (Crow and Stockdale, 1986; Devoto et al., 1996), we observed a lack of signal in mutants in regions known to express *smyhc1* in wild type fish (Fig. 4A). At 3 dpf mutants entirely lacked slow MyHC in adaxial cell-derived SSFs in trunk and tail whereas, depending upon the position along the rostrocaudal axis, siblings had 10-25 SSFs on the surface of each myotome (Fig. 4A). The same mutant larvae, however, retained S58 staining in cardiac, cranial and the somitically-derived anterior hypaxial muscles that extend over the yolk and into the head forming the sternohyoid muscle (Fig. 4A). Moreover, several small groups of fibres known to express *smyhc2* (Elworthy et al., 2008) retained S58 signal in specialised muscle at the dorsal edges of anteriormost somites, the dorsal and ventral edges of the caudalmost somites and in rare cells at the dorsoventral edges and horizontal myoseptum of all somites (Fig. 4A). We conclude that *smyhc1* mutation removes all slow MyHC from the adaxially-derived SSFs, but does not affect Smyhc2 protein accumulation.

To understand the effect of loss of slow MyHC in SSFs we examined other components of the sarcomere (Fig. 4B). Compared to their siblings, the young somites of 24 hpf mutant embryos that entirely lacked slow MyHC showed disruption of thin filament structures, with absence of Z-lines marked by α-actinin and a great reduction in F-actin thin filament arrays (Fig. 4B). F-actin present within the slow fibre region of mutants was concentrated at the vertical myosepta along with α−actinin (Fig. 4B). F-actin also appeared to have irregular periodicity within the mutant myotome, although no α−actinin periodicity was observed (Fig. 4B, yellow boxes). Thus, Smyhc1 appears to be necessary for efficient assembly of both thick and thin filaments within nascent slow muscle fibres.

**Fig. 5:**
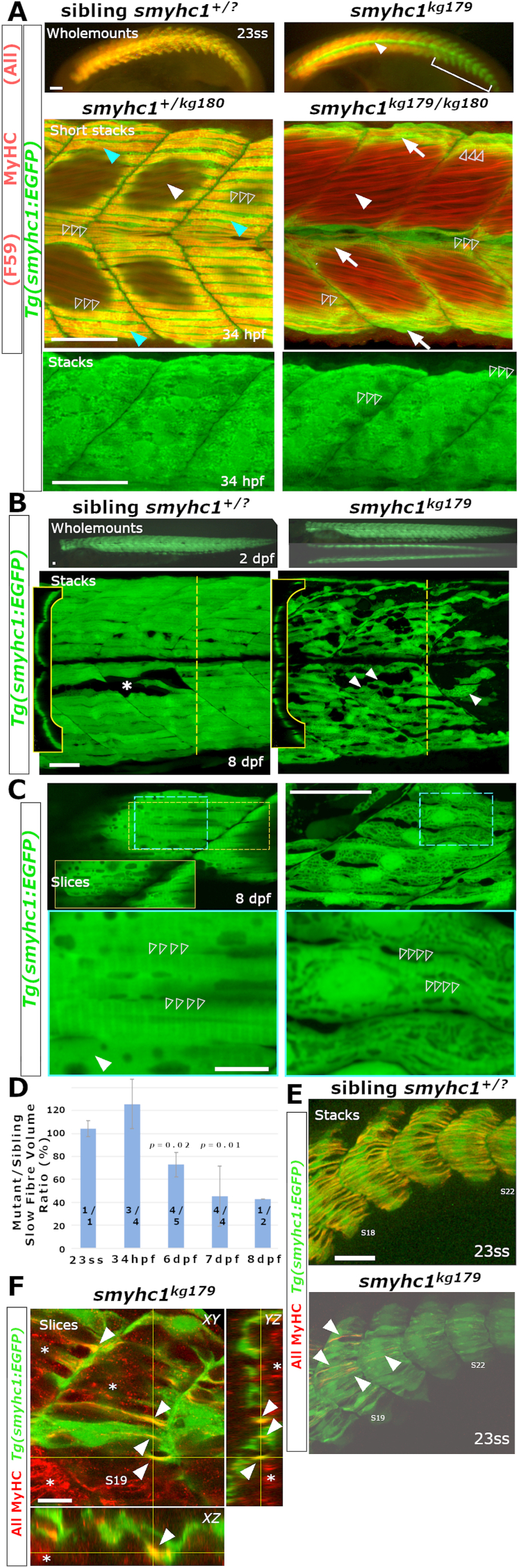
**Survival of SSFs without Smyhc1.** Sibling zebrafish from *smyhc1*^*kg179****/****+*^;*Tg(smyhc1:EGFP)*^*i104*^ male crossed to a female *smyhc1*^*kg180****/****kg180*^ or *smyhc1*^*kg179****/****+*^;*Tg(smyhc1:EGFP)*^*i104*^ mounted dorsal up, anterior to left, shown in live wholemount or confocal maximal intensity projection stacks or slices. **A)** Upper panels: 23ss embryos stained for sarcomeric MyHC revealing absence of signal in slow fibres marked by GFP in mutant. Note the absence of MyHC in muscle pioneers (arrowhead) and slow fibres in tail regions lacking fast MyHC (bracket). Middle panels: Short stacks of somite 19/20 stained for MyHC with F59 showing the presence of a layer of SSFs marked by EGFP (arrows) in both mutant and sibling at 34 hpf. Note the coincidence of strong slow MyHC and EGFP in SSFs (cyan arrowheads) only in the sibling. Dark areas reflect regions of the stack containing underlying fast fibres. The short stack in the mutant was selected to show that MyHC is also weakly detected by F59 in obliquely-orientated fast fibres (white arrowheads). EGFP signal in SSFs of mutant reveals a striated sarcomere-like pattern despite the absence of MyHC (open arrowheads). Lower panels: Magnified full stacks of the same embryos show EGFP in dorsal myotomes of somites 18–20 revealing normal SSF orientation, nuclear positioning and striation (open arrowheads) in mutant. **B)** Live *smyhc1*^*kg179*^;*Tg(smyhc1:EGFP)* mutant and sibling embryos/larvae. Upper panels: Lateral views, with dorsal view below, showing that all SSFs have migrated to the lateral myotome in a 2 dpf mutant. Lower panels: Live 8 dpf larval somites 17–19 showing migrated mutant SSFs detached from the vertical myosepta (arrowheads), compared with a rare fibre defect in the SSF layer in sibling (asterisk). Insets show reduced volume of mutant SSFs in transverse optical sections at the dashed lines. **C)** Single confocal slices of EGFP in SSF layers with blue boxes magnified below. Whereas the sibling had cytoplasm largely filled with myofibrils containing a regular 1.96 ​μm sarcomeric array (open arrowheads), the mutant showed prominent nuclei, a thin disorganized cytoplasm filled with vacuoles between which ran immature and poorly-aligned material with closer striations (open arrowheads). Some fibre regions in sibling were devoid of myofibrils (arrowhead) and contained prominent and extensive vacuoles (Inset yellow box is a slice 5 ​μm more superficial to the dashed yellow box region displaying the abundant vacuolar structures at the myotome surface that are less apparent deeper within the SSF layer. **D**) Quantification of gradually reduced EGFP cell volume of SSFs in *smyhc1*^*kg179*^;*Tg(smyhc1:EGFP)* mutants compared to their siblings at the same age. Mean (± SEM when N allowed, calculated by to include propagation of error in both sibling and mutant measures) of the proportion for scanned segment of at least three somites centred on somite 18. On each column, fraction is number of mutant/sibling fish analysed with identical scan and post-processing parameters. Thus, at 1–2 dpf four embryos had SSFs of similar volume to those in five of their non-mutant siblings whereas, at 6–8 dpf, nine mutant larvae had SSFs about half the size of those in 11 non-mutant siblings. *P*-values above columns represent *t*-test on measured volumes of mutants versus siblings at each age. **E,F)**. Confocal stacks (E) and slices (F) of 2 h-old somites at 23ss showing the emergence of small MyHC-containing myofibrils in mutant SSFs (arrowheads). Crosshairs in F indicate slice planes. Asterisks mark the initiation of deep fast MyHC accumulation in more anterior somites. Bars 50 ​μm (A,B,C upper, E) and 10 ​μm (C lower, F).

Subsequently, however, mutant larvae gained slow MyHC immunoreactivity (Fig. 4C). In *smyhc1*^*kg179*^ mutants at 72 hpf, S58 positive SSFs began to appear at dorsal and ventral somitic extremes and the horizontal myoseptum (Fig. 4C). At each somite extreme between zero and three nascent S58-reactive fibres formed between 1 and 3 dpf. Dual staining with A4.1025, an antibody that recognizes a conserved epitope in the head of all sarcomeric MyHCs, primarily recognized the abundant slow MyHC in SSFs in siblings, and clearly showed that fast fibres had normal sarcomere assembly within the myotome of *smyhc1*^*kg179*^ mutants (Fig. 4C). Loss of staining in SSFs was observed with the F59 antibody, which also detects zebrafish Smyhc1 strongly and other MyHCs more weakly (Fig. 5A). Thus, slow MyHC isoforms distinct from Smyhc1 are recognized by the S58 and F59 antibodies and accumulate in specific fibres within the growing myotome.

### 2.5 Smyhc1 is required for SSF stability, cytoplasmic organisation and growth

To investigate the fate of SSFs that lack slow MyHCs, we next bred the mutants onto the *Tg(smyhc1:EGFP)*^*i104*^ stable transgenic line that labels SSFs due to EGFP expression from the *smyhc1* ATG start codon (Elworthy et al., 2008). Mutants lacked MyHC in almost all slow fibres, but expressed in fast fibres (Fig. 5A). Transheterozygotes of the two *smyhc1* mutant alleles contained slow muscle pioneer fibres at the horizontal myoseptum and normal numbers of migrated SSFs located on the lateral surface of the myotome throughout the body axis (Fig. 5A and B). The SSFs in mutants lacked slow MyHC, but underlying obliquely-oriented fast fibres showed weak immunoreactivity with F59 (but not S58), as previously reported (Fig. 5A; (Devoto et al., 1996)). At 1–2 dpf, SSFs in mutants were initially correctly orientated parallel to the anteroposterior body axis and attached to the vertical myosepta at both anterior and posterior somite boundaries (Fig. 5A lower, B). The volume of slow muscle was unaffected at early stages (Fig. 5D). As mutant larvae matured, EGFP-marked SSFs persisted on the myotome surface until 7 dpf but, by 8 dpf, some had lost contact with one or both vertical myosepta, becoming orientated parallel to underlying superficial fast fibres, and showed a greatly disorganized cell structure and reduced fibre volume (Fig. 5B–D).

The internal structure of SSF cytoplasm was dramatically altered in *smyhc1* mutants. SSFs in wild type and heterozygote siblings had three main sarcoplasmic structures revealed by the distribution of EGFP signal (Fig. 5C). First, an ordered myofibrillar array filling most of the fibre volume. Sarcomeres consisted of narrow bright *I*-band regions and wider and dimmer *A*-band domains with a markedly brighter central *M*-line. Sarcomere length in our live preparations was 1.95 ​± ​0.03 ​μm (mean ​± ​SD, n ​= ​10 sarcomere lengths measured in each of 10 fibres at 8 dpf). Second, the sarcoplasm contained a variety of vacuoles that excluded EGFP, presumably membranous organelles. Numerous smoothly rounded or oblate vacuoles were located near fibre ends and around the nucleus. Less frequent elongated vacuoles appeared to extend anteroposteriorly between myofibrils, some being up to 10 ​μm long. Most SSFs had numerous vacuoles in clusters on their lateral surface, some with complex multilobular forms (Fig. 5C inset). Narrow cytoplasmic bridges extending between vacuoles frequently aligned with *Z*- or *M*-lines. Third, some SSFs appeared to have extended regions containing rather uniform EGFP signal lacking myofibrils but containing vacuoles (Fig. 5C). SSFs in mutants were thin and contained numerous vacuoles that were smaller than those in siblings, even when the SSF was correctly aligned and anchored to both myosepta (Fig. 5C). Moreover, mutant SSFs lacked myofibrillar structures with the characteristic sarcomeric banding. Instead, some regions of cytoplasm had more uniform EGFP signal that exclude vacuoles and appeared to constitute fibrillar structures. The fibrillar EGFP signal was interrupted by narrow dark striations (Fig. 5C), with a striation repeat length of 1.69 ​± ​0.07 ​μm, 13% shorter than the sarcomeres in siblings, and more variable. Mutants did have rare small SSFs at the horizontal myoseptum and dorsal and ventral extremes of the myotome that showed a more normal sarcomeric pattern (Fig. 5C), correlating with the location of residual fibres expressing another slow MyHC isoform (Fig. 4A). Nuclear morphology and positioning in mutants was not distinguishable from that in siblings, in which the nuclei were routinely localized at the lateral surface of the fibre adjacent to dermomyotomal cells and the periderm (Fig. 5C). Quantification of total SSF and MP fibre volume across a series of adjacent somites centred on somite 18 revealed that, on average, mutant fibres had only grown to around half the volume of slow fibres in siblings (Fig. 5D). In summary, without Smyhc1, SSFs have reduced fibre volume, altered cytoplasmic vacuolation, lack mature myofibrils and fail to maintain anchorage to vertical myosepta.

The underlying fast fibres, unlike the SSFs, are not orientated parallel to the anteroposterior body axis (Fig. 4, Fig. 5A). However, SSFs lacking slow MyHC still aligned anteroposteriorly, indicating that they did not convert to a fast myogenic programme. Nevertheless, in young somites, signs of myofibrils with sarcomeric organisation were apparent in the GFP distribution in mutant SSFs (Fig. 5A, open arrowheads). Shortly after mutant SSFs complete their migration to the lateral myotome surface, sarcomeric MyHC was detected in a single myofibril-like structure in many SSFs using the broad-spectrum MyHC A4.1025 antibody (Fig. 5E and F). A similar result was obtained with a second broad-spectrum MyHC antibody (MF20; data not shown). Mutant incross lays were examined for actin structure at 24 hpf with phalloidin-Alexa488. In the absence of Smyhc1 protein, we observed that actin filament organisation was severely defective (Fig. 4A). In wild type siblings, F-actin was organised into sarcomeric thin filament units arrayed at regular intervals along the slow muscle fibre length into myofibrils. In mutant embryo slow fibres, by contrast, overall F-actin signal was reduced and disrupted thin filament organisation was observed. Nevertheless, there were a few fibres in some regions with organised F-actin filaments (Fig. 4B). We conclude that an additional MyHC is expressed in SSFs and can initiate myofibril formation. Nevertheless, without Smyhc1, myofibril growth is severely restricted.

## 3 Discussion

The data presented lead to three new insights into myogenesis. First, we resolve some contradictory findings derived from six *smyhc1* putative null mutant alleles. Second, we find that myosin accumulation is required in order for muscle fibres to grow, thereby illuminating a novel and likely important aspect of muscle fibre growth control. Third, we provide evidence that in zebrafish, as in amniotes, an embryonic fast myosin initiates myofibril formation in nascent slow muscle fibres.

### 3.1 Allelic differences in *smyhc1* mutants

Combining our two new mutant alleles with those previously reported (Li et al., 2020; Whittle et al., 2020), predicted protein-truncating mutations have been made at four positions within Smyhc1 (Fig. 1A). Our early truncations in the first or third coding exons of *smyhc1* show nonsense-mediated mRNA decay, as do the alleles characterised by Li et al. (2020), which also have mutations in the third coding exon. These alleles are therefore likely to be functional null. Indeed, our findings are in most respects congruent with the conclusions of Li et al. (2020). Namely, we observe complete loss of slow MyHC in SSFs at early stages, with slow fibre-specific sarcomere and motility defects and recovery in the 20–30 dpf period as other *smyhc* genes commence expression and generate Smyhc proteins in SSFs. All four *smyhc1* mutants lack detectable slow MyHC protein in fibres that normally express *smyhc1* mRNA, suggesting that, as we show for *smyhc2* and *smyhc3*, despite the presence of the genetically-linked and highly homologous (93–96% amino acid identity) *smyhc2-4* genes, no compensatory upregulation occurs. The similar homozygous phenotypes observed in all early truncation alleles also suggests that, although the guide RNA employed by Li et al. (2020) is perfectly matched not only to *smyhc1* but also to *smyhc2-4* and the mutagenic status of these linked genes was not reported, mutations in *smyhc2* and *smyhc3*, at least, may not be present in the *mb16-18* alleles. Our guide RNAs were designed to sequences specific to *smyhc1*.

One important difference between our findings and those previously published (Li et al., 2020; Whittle et al., 2020) is the lack of significant larval death in our mutants. Although the reason is unclear, we suggest the better survival of our mutants does not arise from allele-specific effects but rather from genetic background or differences in husbandry in the nursery. One such difference is that our fish are fed on rotifers, whereas the larvae carrying *mb16-18* alleles were fed on paramecium (Li et al., 2020). Feeding and survival of *smhyc1*^*stl583*^ homozygotes was not described, but the allele did worsen survival on the background of a semi-dominant truncating allele (Whittle et al., 2020). As Li et al. (2020) showed feeding was variably reduced in their *smhyc1*^*mb17*^ homozygotes, the effect of defective motility on efficiency of food intake could differ with prey motility, size and other parameters.

In contrast to the similarities between the *kg179*, *kg180* and *mb16*-*18* alleles, the *stl583* allele, which is predicted to truncate Smyhc1 towards the end of the myosin S1 head domain, has a number of reported differences. Perhaps most striking is the complete paralysis of *smyhc1*^*stl583*^ homozygotes until 48 hpf (Whittle et al., 2020). Whereas reduced motility at or before 24 hpf allowed selection of embryos mutant for the earlier truncations (Fig. 2B and (Li et al., 2020)), once fast muscle had matured we observed movements in homozygotes for each of our alleles such that they were not readily distinguishable from siblings. Nevertheless, if assayed quantitatively, the early truncated alleles had motility defects that persisted beyond the first week (Fig. 3 and (Li et al., 2020). This reduction in swimming velocity was recovering by 17 dpf (Fig. 3D), an age at which Li et al. (2020) reported increased death of mutant larvae. Indeed, because we found that a continued motility defect became readily apparent by eye when fast myosins were inhibited pharmacologically (Fig. 3B–E), we attribute the effective movement of late embryos and young larvae to fast muscle contraction. One intriguing possibility is that the *smyhc1*^*stl583*^ allele is not null but expresses a partially-functional myosin head fragment that interferes with motility up to 48h hpf. Although *smyhc1*^*stl583*^ homozygotes lack of F59 immunoreactivity, it may be significant that the F59 epitope is in an unmapped region of the myosin S1 head (Crow and Stockdale, 1986) and could therefore be absent in a truncated protein. Use of a head antibody that maps upstream of the *stl583* mutation, such as A4.1025 (Dan-Goor et al., 1990), might reveal the presence of truncated Smyhc1 protein fragments that could have a hypermorphic or neomorphic effect. Such an effect may also explain the variable spinal curves in *smyhc1*^*stl583*^ adults, a phenotype we did not observe and was not reported by Li et al. (2020) in their surviving adults. The possibility of gain of function effects in the *smhyc1*^*stl583*^ allele may reduce the concern expressed by Whittle et al. (2020) over the use of non-specific antisense oligonucleotide approaches to remove toxic gain-of-function MyHCs in human conditions (but see further discussion below).

### 3.2 Muscle fibre growth depends on myosin

The mechanisms coordinating the many cell biological processes required for cell growth are poorly understood. For example, in the century since D’Arcy 10.13039/100004686Thompson formulated the problem in print (Thompson, 1917), how cells match their plasma membrane surface area to the volume of their cytoplasm so as to control cell shape, and how cells generate the appropriate quantity of cytoskeletal elements to support the size and shape of the cell remain mysteries. Skeletal muscle fibres may provide an instructive example of cell volume control because a) their form is constrained to an approximate cylinder by their contractile function, b) their cytoplasm is essentially filled with a specialised actomyosin cytoskeleton and c) they are one of the few cell types that can both greatly increase and decrease in size during adult life. Despite these propitious characteristics for insight into the cell size problem, muscle has been hard to analyse because most fibres are multinucleate. Mutation of murine *MYH4*, encoding MyHC IIB, the major myosin in mouse limb muscles, led to reduced muscle size, but this was accompanied by reduced fibre number, compensatory hypertrophy and an unquantified change in nucleation (Allen et al., 2001). By analysing the early steps in growth of the unusual mononucleate slow fibres of the zebrafish, we reveal the dependence of several other aspects of cell growth on the accumulation of Smyhc1, the major MyHC of these fibres. *Smyhc1* mRNA accumulation precisely parallels the initiation of slow muscle fibre terminal differentiation (Hinits and Hughes, 2007; Hinits et al., 2007). Slow muscle fibres assemble myofibrils prior to their migration (Jana Koth and Simon M. Hughes, unpublished) yet, despite lack of their major MyHC, SSFs migrate and orientate on the surface of the myotome in *smyhc1* mutants in wild type numbers (Fig. 1 and (Li et al., 2020)). No compensatory accumulation of other MyHC isoforms was detected and myofibrils were greatly reduced. Despite attaining normal fibre length, the cross-sectional area, and thus volume, of SSFs fails to increase in the absence of normal myofibril assembly in *smyhc1* mutants (Fig. 5). By 8 dpf, some SSFs become mis-orientated in *smyhc1* mutants. Nevertheless, we and others have observed recovery of SSF morphology, function and presumably cell size in *smyhc1* mutants once Smyhc2 and Smyhc3 become expressed (Li et al., 2020; Whittle et al., 2020). We hypothesise, therefore, that muscle fibre growth control can be likened to the situation with shopping and bags; the quantity of ‘purchased’ cytoskeleton dictates the extent of expansion of the sarcolemmal ‘bags’. How MyHC content or myofibril assembly is assessed and membrane trafficking and/or osmotic balance thereby controlled is of great interest.

### 3.3 An ‘embryonic’ fast MyHC in fish slow muscle fibres

SSFs were present but lacked slow MyHC in *smyhc1* mutants. Nevertheless, muscle fibres in three somitic regions continued to show slow MyHC immunoreactivity in mutants. S58 and F59 immunoreactivity were presented in thin muscle fibres at the dorsal and ventral somitic extremes, the horizontal myoseptum and the posterior end of the tail (Fig. 4) These slow MyHC-expressing somitic fibres match in number, timing and *smyhc2-3* expression the non-adaxially-derived slow fibres described previously (Barresi et al., 2001; Elworthy et al., 2008). In addition, other slow MyHC-immunoreactive cells persisted in mutants in heart and specialised cranial and somite-derived muscles in which other slow MyHC genes, such as *myh6*, *myh7* and *myh7l*, are expressed (Fig. 4A) (Berdougo et al., 2003; Elworthy et al., 2008; Shih et al., 2015; Thisse and Thisse, 2004; Wang et al., 2011). As up-regulation of alternative slow MyHC genes was not observed in *smyhc1* mutants (Li et al., 2020), we conclude that the residual striated myofibril-like organisation in SSFs does not require slow MyHC protein.

By using other anti-sarcomeric MyHC antibodies with broad species and isoform cross-reactivity (namely, A4.1025 and MF20), however, we observed striated MyHC immunoreactivity in the SSFs of *smyhc1* mutants. This persistent MyHC was located in myofibril-like structures that also contained F-actin arrays and were compact enough in some regions to exclude the cytoplasmic EGFP that marked the SSFs, making the myofibril-like structures visible in live larvae. Such structures had reduced sarcomere length, as reported in the residual actin and α-actinin-containing structures in other *smyhc1* mutants analysed (Li et al., 2020; Whittle et al., 2020). It is possible that the gene responsible for this MyHC accumulation in SSFs lacking Smyhc1 is an *myhz1*, which may also be expressed in adaxial SSF-precursors from the initiation of their terminal differentiation (Hinits and Hughes, 2007; Hinits et al., 2007). Thus, the ability of SSFs in *smyhc1* mutants to persist and recover function once alternative slow MyHCs start to express around ∼20 dpf may reflect the resumption of a developmental progression prevented by the lack of Smyhc1. We hypothesise that, in the presence of Smyhc1, this progression would allow seamless maturation of sarcomere length and expansion of myofibril size in the early embryo SSFs.

There are some similarities between the defective SSFs in *smyhc1* mutants and those arising after Mef2 knockdown (Hinits and Hughes, 2007). Reduced Mef2 activity leads to failure to incorporate Smyhc1 into myofibrils, leaving a ‘dumbbell’ morphology fibre with a single thin myofibril and a cloud of unincorporated Smyhc1 at each end (Hinits and Hughes, 2007). In the absence of Smyhc1, a similar nascent myofibril structure perdures. As Mef2 activity is not required for *smyhc1* expression in SSFs, it seems that both Smyhc1 and at least one other Mef2-dependent protein are required for growth of myofibrils. An obvious possibility is the embryonically-expressed fast MyHC present in *smyhc1* mutants.

The presence of a distinct MyHC isoform in nascent fibres in zebrafish is reminiscent of the embryonic MyHC, expressed from the *MYH3* gene, in nascent mammalian fibres irrespective of the eventual mature fibre type (Condon et al., 1990; Karsch-Mizrachi et al., 1989; Schiaffino et al., 2015; Whalen et al., 1981). Knockout of murine *Myh3* leads to reduced viability, muscle weight and fibre cross-sectional area and a relative increase in slow myogenesis, accompanied by knock-on effects on muscle stem cells (Agarwal et al., 2020). Viable *Myh3* mutants are compensated by up-regulation of other fast MyHC genes (Agarwal et al., 2020). Our finding of reduced fibre size when Smyhc1 is missing followed by compensation when additional Smyhc proteins accumulate suggests that loss of either of two MyHC isoforms in nascent myofibres may have similar effects. In this light, it is interesting that point mutations in the fast-class *MYH3* gene cause several arthrogryposes (Toydemir et al., 2006). As similar mutations in the slow-class *smyhc1* gene cause defects in zebrafish (Whittle et al., 2020), it will be interesting to determine whether mutation of the remaining MyHC in SSFs has similar effects.

## 4 Methods

### 4.1 Zebrafish lines and maintenance

All lines used were reared at King's College London on a 14/10 ​h light/dark cycle at 28.5 ​°C with adults kept at 26.5 ​°C, with staging and husbandry as described in (Westerfield, 2007). AB stocks were maintained by breeding at least ten pairs at each generation. Embryos/larvae were reared at 28.5 ​°C in the dark, except for periods outside the incubator until 6 dpf when rotifer feeding commenced. *Smyhc1*^*kg179*^ and *smyhc1*^*kg180*^ mutant alleles on AB background were genotyped by High Resolution Melt Analysis (HRM), followed by sequencing using primers indicated (Table 1). Briefly, HRM primers amplified DNA fragments of 107 bp, 103 bp, 115 bp and 113 bp and sequencing primers amplified DNA fragments of 603 bp, 599 bp, 280 bp and 278 bp *smyhc1*^*+/+*^, *smyhc1*^*kg179*^*, smyhc1*^*+/+*^ and *smyhc1*^*kg180*^ alleles, respectively. *Tg(smyhc1:EGFP)*^*i104*^ was back-crossed onto *smyhc1*^*kg179*^. All experiments were performed on zebrafish derived from F3 or later generations, in accordance with licences held under the UK Animals (Scientific Procedures) Act 1986 and later modifications and conforming to all relevant guidelines and regulations.

**Table 1:** Primers for HRM and sequencing at *smyhc1* target loci and for ISH probe synthesis.

*Smyhc1 – exon 2* (*kg180*)	Sequence
Forward HRM (amplicon size 107 bp)	5’-CGCAAGTCTGACAAGGAGC-3’
Reverse HRM	5’-GTGATGGAGGCTTTGACGTAC-3’
Forward Sequencing (amplicon size 603 bp)	5’-CCTGTGCTGTTCCTTTTCTCA-3’
Reverse Sequencing	5’-CCATGAGACTGTGTTGGCTG-3’
***Smyhc1 – exon 4* (*kg179*)**	
Forward HRM (amplicon size 115 bp)	5’-TCTGTGTCACTGTCAACCCA-3’
Reverse HRM	5’-AGTTCTCACCTGACAGCAT-3’
Forward Sequencing (amplicon size 280 bp)	5’-TGAGTGATGAACGTTGAGCC-3’
Reverse Sequencing	5’-AAATGAGGGAAGTTTTGTGCAT-3’
***Smyhc1* in situ hybridisation**	
Forward (T7)	5'TAATACGACTCACTATAGGGAGAGTAAGAACCAAAGAGCTTCCATG 3'
Reverse (T3)	5’GGATCCATTAACCCTCACTAAAGGGAATGAAATCTTGTGTTTGTCAGACC3’

### 4.2 Generation of *smyhc1* mutants

*Smyhc1* mutants were generated by targeting the sequence 5′-CCCAAACTCGTATTTTTGACATG-3’ (exon 2) and 5′-CCAGTGTACGATTCCTCTGTGGT-3’ (exon 4) of *smyhc1.* CRISPR guide RNAs were selected with CRISPR Direct minimizing potential off-targets (Naito et al., 2015). Optimised flanking primers (Integrated DNA Technologies) creating a ∼120 bp PCR product for HRM (20 bp each, Tm 60 ​°C) and 200–700 bp product for DNA sequencing were selected for each gRNA with Primer 3 software (Koressaar and Remm, 2007). CRISPR oligonucleotides (Table 2) were annealed, ligated into BsaI-digested pDR274 (Addgene), plasmid DNA purified, sequenced, digested with DraI and the 284 bp fragment gel-purified and used to synthesise gRNA with T7 RiboMAX large scale RNA production kit (Promega). gRNA was phenol/chloroform purified, ethanol precipitated, quantified by gel and Qubit (Invitrogen), aliquoted in 5 ​μl samples at 2 ​μg/μl and stored at −80 ​°C.

**Table 2:** Oligonucleotides to anneal to target *smyhc1* for mutation.

gRNA description	Oligonucleotides for insertion into pDR274
gRNA KO1 (*kg180)*	Oligo 1: 5’-TAGGTGTCAAAAATACGAGTTT-3’
	Oligo 2: 3’- ACAGTTTTTATGCTCAAACAAA-5’
gRNA KO2 (*kg179*)	Oligo 1: 5’-TAGGCACAGAGGAATCGTACAC-3’
	Oligo 2: 3’- GTGTCTCCTTAGCATGTGCAAA-5’

HRM-selected AB wild-type fish were DNA sequenced over the target loci to avoid polymorphisms, crossed and the resulting embryos injected with 1 ​nl containing 40 ​pg (gRNA2) or 80 ​pg gRNA1, 300 ​pg *sp*Cas9 protein with 0.03% rhodamine dextran to select injected embryos. Ten 48 hpf larvae were analysed by HRM to verify mutagenesis, their F0 siblings grown to adulthood and outcross F1 progeny analysed for transmission by HRM. Mutant loci of F1 heterozygotes were sequenced to identify F0s transmitting mutations of interest, F1 siblings grown to adulthood and F1 heterozygotes identified by HRM and sequencing of fin-clip DNA. Subsequent generations were bred by outcross to wild-type AB selected as non-polymorphic at the target locus.

### 4.3 RNA extraction and RT-qPCR

Protocol was previously described (Ganassi et al., 2018; Kelu et al., 2020). Pools of five (5 dpf) or 10 (1, 2 dpf) embryos were snap-frozen in liquid nitrogen with minimal liquid. Samples were sonicated on ice using 350 ​μL RNeasy Lysis Buffer (Buffer RLT) from RNeasy Mini Kit (Qiagen), lysates treated with RQ1 RNase-free DNase (Promega) and column-purified using RNeasy Mini Kit (Qiagen) according to manufacturers' instructions. Purified total RNA (500 ​ng) was subsequently reverse transcribed using High-Capacity cDNA Reverse Transcription Kit (Applied Biosystems) with both random and oligo-dT primers (Invitrogen) and RNase inhibitor (NEB). For RT-qPCR, technical triplicates were performed on 5 ​ng of cDNA using Takyon Low ROX SYBR 2X MasterMix blue dTTP (Eurogentec) using ViiA™ 7 Real-Time PCR System (Thermo Fisher Scientific) in a 10 ​μL reaction using primers listed (Table 3). Specific primers were designed to target unique regions in the 5′-UTR *of smyhc1-3* transcripts using Primer-BLAST (Ye et al., 2012) (Supplementary Fig. 1A). An additional primer set targeting 3′-UTR of *smyhc1* was previously described (Li et al., 2020) (Supplementary Fig. 1), but with a modification in the reverse primer to account for a SNP. *eef1a1l1* was used as a housekeeping gene as its expression is high and stable during zebrafish development (McCurley and Callard, 2008). ΔCt was calculated by subtracting the triplicate mean Ct value of *eef1a1l1* from that of the target gene. ΔΔCt of each target gene was then calculated by subtracting the mean ΔCt of target and housekeeping samples, and relative gene expression calculated as 2^−ΔΔCt^ (Livak and Schmittgen, 2001), followed by normalisation to the mean of 1 dpf motile control samples.

**Table 3:** Primers for RT-qPCR.

RT-qPCR primers	
Forward *eef1a1l1* (amplicon size 140 bp) (Kelu et al., 2020)	5'-AGCAGCAGCTGAGGAGTGAT-3'
Reverse *eef1a1l1* (Kelu et al., 2020)	5'-CCGCATTTGTAGATCAGATGG-3'
Forward (F1) *smyhc1* (5′UTR; amplicon size 240 bp)	5'-CAAACACTGCATCCAAAGCCAA-3'
Reverse (R1) *smyhc1* (5′UTR)	5'-CTTGTCAGACTTGCGCAGGAA-3'
Forward (F2) *smyhc1* (3′UTR; amplicon size 220 bp) (Li et al., 2020)	5'-TGAAGAGGCTGAGGAACAGG-3'
Reverse (R2) *smyhc1* (3′UTR) **(∗A/G SNP)**	5'-AGAACCAGTACTT**A**AACATGGC-3'
Forward *smyhc2* (5′UTR; amplicon size 87 bp)	5'-TGGCGAGGCACACTTTCATC-3'
Reverse *smyhc2* (5′UTR)	5'-TTACGTAAGAACGGAGCCGC-3'
Forward *smyhc3* (5′UTR; amplicon size 159 bp)	5'-TTTTTGAGCGCACTGAACCAT-3'
Reverse *smyhc3* (5′UTR)	5'-TCATGTCAAAGGGGCGAGTT-3'

### 4.4 Imaging, in situ mRNA hybridization and immunodetection

ISH and immunodetection were performed as described (Thisse and Thisse, 2004). Digoxigenin-labelled probes for *smyhc1* was made by PCR from 1 dpf cDNA using listed primer pairs (Table 1) with an added T7 polymerase binding site on the reverse primer. For in situ imaging, embryos were immersed in glycerol and images collected on a Leica MZ16F with LED light attachment, Olympus DP70 camera and DP Controller software. Primary mouse monoclonal antibody supernatants of A4.1025 1:5 (Dan-Goor et al., 1990), F59, S58 (1:5 (Crow and Stockdale, 1986; Devoto et al., 1996);) and EB165 (neat (Blagden et al., 1997);), against all sarcomeric, slow and fast MyHC respectively, were detected with Goat anti-Mouse IgG-Alexa555 (Invitrogen, A21422) and Goat anti-Mouse IgA-FITC (Serotec, 5104-3410F), respectively. Phalloidin-Alexa555 (Invitrogen, A34055) For confocal imaging unfed embryos/larvae (tricaine-anaesthetised if live) were mounted in glycerol, Citifluor (Agar) or 0.8–1% low melting point agarose and data collected on a LSM Exciter microscope (Zeiss) equipped with 63x/1.1oil, 40x/1.1Corr or 20x/1.0W objective and subsequently analysed using Fiji (NIH, ImageJ2 2.9.0/1.53t) or ZEN 2009 (Zeiss) software. Volume measurements were performed on confocal stacks (20x/1.0W objective, 0.5 or 1x zoom, pinhole 1 Airy, 1 ​μm slice thickness) of fixed (23ss) or live (other stages) fish, with all individuals within each lay scanned under identical microscope settings and analysed with the Fiji ‘3D Objects Counter’ plugin under constant Threshold.

### 4.5 Adult fish analysis

Siblings (4 mpf) from single heterozygote in-crosses were anaesthetised with tricaine (Sigma Aldrich), sexed, blotted dry and weighed on an Ohaus YA102 balance, standard length measured against a ruler and fin-clipped for sequence genotyping. Weights and lengths were compared by unbalanced two way (genotype and sex) ANOVA with Tukey post hoc tests (GraphPad PRISM 8).

### 4.6 Swimming velocity test

Chorions were removed from 1 dpf siblings from single heterozygote in-crosses. Individual embryos were analysed for presence or absence of movement in response to touch stimulus. At 2 dpf and later, embryos/larvae from 3 to 4 lays were stimulated by touch using a needle and video recorded using Leica MZ16 with Olympus DP70 camera and DP Controller at 30.53 frames.s^−1^ before and after treatment with 50 ​μM BTS for 10 ​min. Fish velocity measured using Tracker (https://physlets.org/tracker/). Embryos/larvae were retrospectively genotyped. Statistics were analysed in RStudio 1.4.1103 separately for control Fish Water and BTS-treated fish with factors Age, Genotype, Lay and Age∗Genotype interaction. Velocity measurements were log-transformed to ensure equal variance (homoscedasticity) based on diagnostic plots of unexplained variance. The Akaike information criterion was used to select the most informative ANOVA, which was two way ANOVA on Age and Genotype with interaction. No effect of Lay was detected. Graphpad Prism was used for one way ANOVA.

## Author contributions

HTH performed the experiments and wrote an initial draft. JJK performed experiments. SMH contributed to study design, data analysis and writing. JO and SMH initiated the project and obtained the funding.

## Declaration of competing interest

All authors declare they have no financial or competing interests.

## Data Availability

Data will be made available on request.
